# Metabolism and Signaling of Plant Mitochondria in Adaptation to Environmental Stresses

**DOI:** 10.3390/ijms231911176

**Published:** 2022-09-23

**Authors:** Pedro Barreto, Alessandra Koltun, Juliana Nonato, Juliana Yassitepe, Ivan de Godoy Maia, Paulo Arruda

**Affiliations:** 1 Departamento de Ciências Químicas e Biológicas, Instituto de Biociências, Universidade Estadual Paulista, Botucatu 18618-970, Brazil; 2 Genomics for Climate Change Research Center, Universidade Estadual de Campinas, Campinas 13083-875, Brazil; 3 Departamento de Genética e Evolução, Instituto de Biologia, Universidade Estadual de Campinas, Campinas 13083-862, Brazil; 4 Embrapa Agricultura Digital, Campinas 13083-886, Brazil; 5 Centro de Biologia Molecular e Engenharia Genética, Universidade Estadual de Campinas, Campinas 13083-875, Brazil

**Keywords:** *Arabidopsis thaliana*, retrograde signaling, alternative respiration, uncoupling proteins, abiotic stresses, hypoxia signaling, crop improvement, plant performance

## Abstract

The interaction of mitochondria with cellular components evolved differently in plants and mammals; in plants, the organelle contains proteins such as ALTERNATIVE OXIDASES (AOXs), which, in conjunction with internal and external ALTERNATIVE NAD(P)H DEHYDROGENASES, allow canonical oxidative phosphorylation (OXPHOS) to be bypassed. Plant mitochondria also contain UNCOUPLING PROTEINS (UCPs) that bypass OXPHOS. Recent work revealed that OXPHOS bypass performed by AOXs and UCPs is linked with new mechanisms of mitochondrial retrograde signaling. AOX is functionally associated with the NO APICAL MERISTEM transcription factors, which mediate mitochondrial retrograde signaling, while UCP1 can regulate the plant oxygen-sensing mechanism via the PRT6 N-Degron. Here, we discuss the crosstalk or the independent action of AOXs and UCPs on mitochondrial retrograde signaling associated with abiotic stress responses. We also discuss how mitochondrial function and retrograde signaling mechanisms affect chloroplast function. Additionally, we discuss how mitochondrial inner membrane transporters can mediate mitochondrial communication with other organelles. Lastly, we review how mitochondrial metabolism can be used to improve crop resilience to environmental stresses. In this respect, we particularly focus on the contribution of Brazilian research groups to advances in the topic of mitochondrial metabolism and signaling.

## 1. Introduction

Mitochondria are classically referred to as the cell powerhouse due to their central function in hosting the oxidative phosphorylation (OXPHOS) and tricarboxylic acid cycle (TCA) machinery [1]. Together, OXPHOS and TCA represent the following major functional roles of mitochondria: energy production and support for the biosynthesis of metabolic intermediates. These features make this machinery central to diverse biological outcomes, including proliferation, differentiation, and adaptation to stress [2]. Since mitochondria are rarely considered to dictate commands or provide signals to change biological outcomes, changes in mitochondrial metabolism can occur due to alterations in nuclear gene expression. However, when a cell commits to a process such as proliferation or differentiation without adequately functioning mitochondria, the cell would likely undergo a metabolic crisis, possibly resulting in cell death or senescence [1,2,3]. In this scenario, properly functioning mitochondria can be seen as an early checkpoint before cells commit to any developmental or stress-response processes. A notable example occurs within the first minutes of the plant life cycle in which mitochondria are fully active almost immediately upon seed rehydration [4]. However, it is implausible that plants begin their life cycle with poorly functioning mitochondria.

Mitochondria likely originated from α-proteobacteria, which developed an endosymbiotic relationship with the host archaeon [5]. The nature and benefit of this symbiosis are fervently debated. A long-held belief is that the α-proteobacteria initially provided ATP or detoxified reactive oxygen for their archaeon host. However, mitochondria possess abundant electron donors, which can provide electrons to oxygen to form reactive oxygen species (ROS) [1,6]. Therefore, mitochondria may toxify more than they detoxify oxygen. Another possible explanation for the original symbiosis, as described in the “hydrogen hypothesis” [7], is that the α-proteobacteria and its host provided important metabolites for each other. In this scenario, eukaryotes may have evolved from a metabolic relationship between α-proteobacteria and a methanogenic archaeon. During evolution, the mitochondrion lost control over most of its genome, and a functional transfer of mitochondrial genes to the nuclear genome occurred. Consequently, most of the ~2000 proteins that compose a single mitochondrion are nuclear-encoded [8]. Therefore, mitochondria and the nucleus must coordinate to achieve adequate cellular functions in various cellular processes. Independent of the evolutionary theory regarding mitochondrial origins, we can infer that mitochondrial communication with other cell compartments is among eukaryotes’ most ancient forms of signaling.

In this review, we revisit aspects that concern fundamental mitochondrial processes in OXPHOS and how plant mitochondria sense and adapt to changes in the cellular environment. We further discuss how the signals provided by mitochondria are transduced to other cell compartments in the context of recent advances in plant mitochondrial signaling. Lastly, we discuss how studies on mitochondrial biology can help develop plants better adapted to adverse environmental conditions, with a particular focus on the contributions of Brazilian research groups.

## 2. Plant Oxidative Phosphorylation Bypasses: Importance for Mitochondrial Signaling and Stress Response

**Figure 1:**
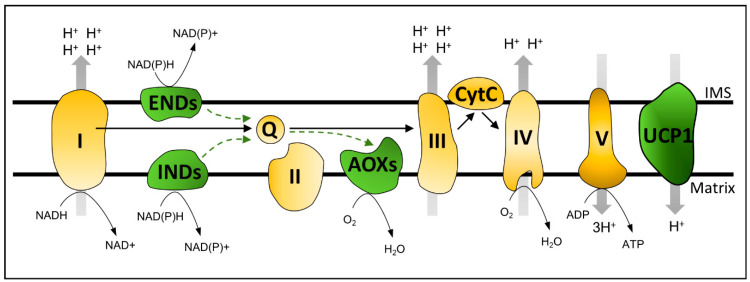
Plant mitochondrial oxidative phosphorylation (OXPHOS). Schematic representation of the canonical mitochondrial oxidative phosphorylation machinery (yellow) composed of the four multi-subunit complexes of the mitochondrial electron transport chain (Complex I–IV) and two intermediary substrates, namely, ubiquinone (Q) and cytochrome *c* (CytC). These components generate an electrochemical gradient between the mitochondrial inner membrane space (IMS) and the matrix. Protons flow back to the matrix via Complex V to produce ATP. Bypasses of the canonical OXPHOS (green) include INTERNAL (INDs) and EXTERNAL (ENDs) NAD(P)H DEHYDROGENASES, ALTERNATIVE OXIDASES (AOXs), and UNCOUPLING PROTEINS (UCPs). Protein sizes are not scaled.

In addition to TCA cycle-related enzymes, plant mitochondria contain standard oxidative phosphorylation machinery composed of four electron transport chain (ETC) complexes (I–IV) and ATP SYNTHASE (Complex V). Together, TCA and ETC components catalyze mitochondrial oxidative phosphorylation with the following proton pumping sites: Complexes I, III, and IV [6]. The electrochemical gradient is used by ATP SYNTHASE to produce ATP from ADP and Pi (Figure 1). Proton pumping to the inner membrane space and the consequent increase in mitochondrial membrane potential (ΔΨm) by the ETC must be coupled to the dissipation of the electrochemical gradient by Complex V. This balance remains relatively stable. However, limited fluctuations in both factors can occur, reflecting normal physiological activity or adverse environmental conditions [9]. Under a high electrochemical proton gradient, the ETC may induce mitochondrial dysfunction, possibly via ROS formation. Under such circumstances, mitochondria can use specific mechanisms, such as divalent ion (e.g., Ca^+2^) transporters [9] or energy bypass proteins, to remove excess reductants, thereby restoring mitochondrial ΔΨm [3,6]. In this section, we review how OXPHOS energy bypasses, such as UNCOUPLING PROTEINS (UCPs), ALTERNATIVE OXIDASES (AOXs), and ALTERNATIVE NAD(P)H DEHYDROGENASES (NDs), serve as tools for studying mitochondrial signaling and stress response.

### 2.1. UNCOUPLING PROTEINS

The UNCOUPLING PROTEINS are mitochondrial components that are thought to confer metabolic flexibility to respiration by dissipating mitochondrial ΔΨm, in a fatty-acid-dependent manner, uncoupled from ATP synthesis [10] (Figure 1). *Arabidopsis thaliana* (hereafter referred to as *Arabidopsis*) UCP1 occupies approximately 1% of the mitochondrial inner membrane (MIM) area [11]. Distinct from AOXs and NDs bypassing proton pumps, UCPs dissipate the proton gradient downstream of the electron transport chain [10,12] (Figure 1). Anibal Vercesi’s group led to the discovery and biochemical characterization of the plant UCP1 in the 1990s [10]. An extensive biochemical characterization by Vercesi’s group and others confirmed the role of plant UCP1 as an H^+^ transporter uncoupled from ATP synthesis [12]. Soon after its discovery in *Solanum tuberosum*, an ortholog of *Arabidopsis* UCP1 was identified in a joint effort between Vercesi’s and Arruda’s group at the State University of Campinas [13]. The work on UCP1 continued in Brazil and is currently led by Ivan Maia’s and Paulo Arruda’s groups, in which model and crop plants are under study. However, a recent study suggests that *Arabidopsis* UCP1 and UCP2 function as metabolite transporters rather than respiration uncouplers [14]. Despite the relevance of this study, the functional role of UCP1 is much more complex and needs to be considered in the cell, organ, and environmental context instead of simple systems, such as liposomes. Regardless, experimental data for the in vivo biological function of UCP1 are still lacking, and this is among the top questions to be answered in plant mitochondrial biology [6]. Biochemical characterization together with the latest findings and hypotheses regarding plant UCPs were comprehensively reviewed in [12,15].

**Figure 2:**
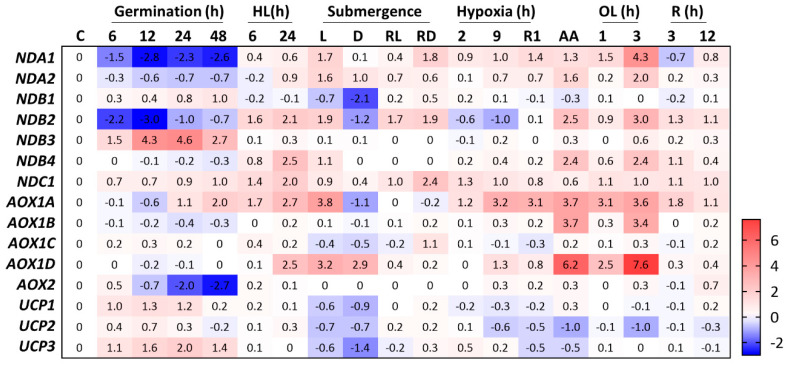
Relative expression of the plant OXPHOS bypasses during germination, abiotic stresses, and chemical treatments. Gene expression data were obtained from the GENEVESTIGATOR database [18]. Original datasets used to build this heatmap were obtained from the Gene Expression Omnibus (GEO) within the National Center for Biotechnology Information (NCBI) database under the following accessions numbers: germination (GSE30223), high light (HL, GSE111062), waterlogging (SRP120444), hypoxia (GSE9719), antimycin (AA, GSE36011), oligomycin (OL, GSE38965), and rotenone (R, E-MEXP-1797). Germination, high light, hypoxia, oligomycin, and rotenone treatments are displayed as hours (h) under the respective treatment. Relative expression is expressed as the log_2_ fold-change compared to untreated plants (C) within each condition. Data are not comparable between distinct experiments. For definitions of gene symbols and for a complete description of the gene names, please see Table S1. L: light, D: dark, RL: recovery of light-submerged plants, RD: recovery of dark-submerged plants, R1: 1 h reoxygenation.

Similar to other mitochondrial OXPHOS bypasses, UCPs decrease the pressure on the electron transport chain under abiotic stress by facilitating mitochondrial ΔΨm dissipation under conditions of high adenylate charge [16]. Interestingly, high-light stress was recently shown to induce UCP1 protein accumulation in *Arabidopsis aox1a* antisense lines but not in the wildtype (WT) plants [17]. In addition, GENEVESTIGATOR [18] analysis of publicly available transcriptomes did not suggest that any of the *Arabidopsis UCPs* are induced under this condition in WT plants (Figure 2). These results suggest that UCP1 plays a role in situations where an excess of reducing power occurs at the electron transport chain, similar to AOXs and NDs, but specifically when AOX is absent. Compared to WT and *ucp1* knockdown lines, plants overexpressing UCP1 perform better under saline, oxidative, hyperosmotic, hypoxia, and water stress conditions [15,19,20,21]. In addition, UCP1 was recently shown to be important for plants to tolerate reductive stress caused by dithiothreitol (DTT) [22]. Arabidopsis seedlings grown in the presence of DTT displayed a marked inhibition of primary root growth in both *ucp1* and *aox1a* knockdown lines, suggesting that low-molecular-weight thiols might donate electrons to the mitochondrial ETC, causing overreduction of its components, which in turn results in insufficient substrate oxidation rates. Thus, the observed phenotypes might be explained by a reduced ETC capacity in the mutant lines [22]. In keeping with this hypothesis, impaired ETC that leads to reduced NADH oxidation is believed to cause the diminished photorespiratory capacity of the *ucp1* mutant [23]. However, under reductive stress, no alterations in NAD content were found in the *ucp1* mutant lines after exposure to DTT, which contrasts with the results observed for *aox1* plants, suggesting distinct mechanisms of adaptation in these lines. In this scenario, the presence of a compensatory mechanism upon other *UCPs* when a specific isoform is mutated should be considered [24]. A striking feature of *UCP1* is that it exhibits relatively constant expression under a variety of developmental stages and in response to different mitochondrial inhibitors, such as antimycin (AA) [25], oligomycin [26], or rotenone [27] (Figure 2). Significant changes in *UCP1* expression appear to correlate with increased metabolic and mitochondrial activity in tissues and developmental stages, such as germination (Figure 2) and flower development [28,29]. This seems counterintuitive because UCP1 function is thought to be in energy dissipation; however, because UCP1 is a major component of the MIM, it may simply be a consequence of increased mitochondrial content. In this context, alterations in UCP1 content seem to affect germination when the seeds face adverse conditions [24,30], although the precise mechanisms or in vivo function of UCP1 during this developmental stage have not been investigated thus far. The association of *UCP1* expression with reproductive organs/tissues was further investigated using transgenic tobacco plants harboring the promoter regions of *Arabidopsis UCP1* and *UCP2* fused with the GUS reporter [30]. Consistent with transcriptome data [29], *UCP1* promoter activity was markedly increased in both male and female reproductive organs. The *ucp1* mutant line [23] and double mutant lines for different *UCP* family members [24] exhibited strong hydrogen peroxide (H_2_O_2_) accumulation in male and female organs. In addition, alteration in the expression of several regulators of reproductive development was observed in these lines when grown under regular conditions [24,30]. These changes resulted in the reduced seed and silique size observed in double mutant lines [24]. The opposite effect was observed in tobacco lines overexpressing *Arabidopsis UCP1* [21]. These lines maintained higher fertility and seed production when grown under drought stress and increased seed size under normal growth conditions. Although it has been hypothesized that this might be a consequence of the altered respiratory capacity necessary for flower development, particularly in anthers and pollen, future studies considering UCP1 function in vivo are needed.

### 2.2. ALTERNATIVE OXIDASES and ALTERNATIVE NAD(P)H DEHYDROGENASES

An interesting feature of plant mitochondria is the respiratory capacity in the presence of rotenone (a complex I inhibitor) and cyanide (a complex IV inhibitor), which inhibit mammalian mitochondrial respiration. In fungi, plants, and some metazoans, two key steps of the mitochondrial respiratory chain, namely, ubiquinone reduction and ubiquinol oxidation, differ from mammals, as the steps involve the bypassing enzymes NDs and AOXs [6]. ALTERNATIVE NAD(P)H DEHYDROGENASES can functionally replace the NADH oxidizing activity of Complex I, transferring electrons from NADH directly to ubiquinone without a proton pump, while AOXs can be a functional substitute for Complexes III and IV (as they can transfer electrons from a ubiquinol pool directly to oxygen) [6,31] (Figure 1). Studies in the plant mitochondrial retrograde signaling area were boosted by the discovery that chemically induced mitochondrial dysfunction could induce the expression of *AOXs* [32]. Due to their reported co-expression, the *AOX* isoforms and corresponding *NDs* are suggested to act as cooperative functional units [33,34,35,36,37,38]. Therefore, this section discusses both AOXs and NDs, focusing on mitochondrial signaling and the integration of cellular metabolism.

#### 2.2.1. ALTERNATIVE OXIDASES

ALTERNATIVE OXIDASES are cyanide-insensitive ubiquinol oxidases located in the mitochondrial inner membrane that can catalyze oxygen reduction to water without pumping protons [6] (Figure 1). Even though AOXs reduce energy efficiency, their activity is part of several physiological conditions aimed at conferring metabolic flexibility and stress tolerance, in addition to growth maintenance in balance with plant resource availability. ALTERNATIVE OXIDASE activity usually increases under stress conditions, whereas the partitioning of electrons between the cytochrome and the alternative pathway is variable. Intriguingly, even without adverse environmental conditions, energy flow through the alternative pathway accounts for 10–50% of total respiration [39]. Interestingly, the amount of AOX protein was only 4% of that of Complex IV proteins (693 vs. 17,243 copies per mitochondrion) [11]. The mitochondrial alternative oxidase pathway has been extensively assigned as a sink for excess reductants that are generated in the chloroplast under high-light stress. Thus, the pathway may reduce photoinhibition [39]. The association between the alternative pathway and photosynthesis seems unequivocal, as robust studies have provided solid in vivo physiological [40,41] and molecular evidence [42]. In this context, we recommend reading Vanlerberghe et al.’s review on ALTERNATIVE OXIDASES and photosynthesis relationships [43].

Arabidopsis contains the following *AOX* genes: four *AOX1* (*a*–*d*) and one *AOX2* [44], but *AOX1a* is the most highly expressed isoform throughout *Arabidopsis* development. The isoforms *AOX1a* and *AOX1d* have a clear induction pattern in response to stresses [45,46]. Chemical treatments such as AA [25] and oligomycin [26] induce both *AOX1a* and *AOX1d* but not the other isoforms (Figure 2). It is also interesting to note that, during germination [47], *AOX1a* and *AOX2* are up- and downregulated, respectively, while there are no alterations in the other isoforms (Figure 2). Here, we focus on the highly investigated AOX1a, a major component of the mitochondrial proteome compared with other AOXs [11], even though recent findings demonstrate that AOX1d can compensate for the lack of AOX1a [46,48].

An emerging topic related to AOXs is their role in hypoxia. The regulation of AOXs in response to altered oxygen levels is not a novelty since it was reported that soybean plants primed with anoxia treatment resulted in further protection against H_2_O_2_-induced cell death, which seemed to involve AOXs [49]. In addition to performing ROS scavenging, higher levels of AOX produced during low oxygen concentrations prepare the cells to cope with the electron overflow that occurs during reoxygenation [49]. Analysis of publicly available transcriptomic data through GENEVESTIGATOR [18] reveals a variety of *AOX* expression patterns in microarray and transcriptomic data of *Arabidopsis* plants subjected to distinct degrees of hypoxia or submergence. Expression values and patterns differ when distinct *Arabidopsis* accessions and tissues are compared; thus, quite contrasting results have been reported in the literature. For example, a detailed time-course analysis of *Arabidopsis* roots subjected to 4% O_2_ for up to 48 h revealed no change in *AOX1a* transcript levels [50]. In the opposite direction, pronounced induction was observed for *AOX1a* (16-fold upregulated) in seedling roots treated with 0.1% O_2_ for 5 h [51]. Additionally, the *AOX1a* transcript was markedly upregulated in the polysomes of hypoxia-treated seedlings [52] (Figure 2). The difference in these results might be due to the degree of hypoxia imposed on the plants or treatment duration. The results also indicated a possible role for AOX1a during flooding [53]. In this regard, whole plants submerged in the dark showed twofold repression of *AOX1a* expression in leaves, whereas an eightfold induction was observed in light-submerged plants compared to the untreated control (Figure 2). Submerged plants ultimately suffer from a shortage in cellular oxygen availability due to impaired gas diffusion, an outcome that is not limited to oxygen but also includes ethylene, CO_2_, and nitric oxide (NO) [54]. This may help to explain the more pronounced *AOX1a* expression patterns observed in submerged plants in the presence of light. Although conflicting and possibly resulting from the technical approaches used to impose hypoxia or submergence, this evidence allows us to infer that the *AOX1a* transcript level is not stable under hypoxia. In general, it should be expected that AOX1a-dependent respiration decreases during hypoxia, as it reduces respiration efficiency, but recent results showing the involvement of AOX in NO signaling may help us to understand why AOX1-dependent respiration would instead increase upon hypoxia.

Nitric oxide production and its different metabolic and signaling functions in plants, especially concerning mitochondrial components, have become an emerging field within the last decade [55]. Plant mitochondria produce high levels of NO under low oxygen through Complex III and IV in the electron transport chain [55,56]. Interestingly, NO can inhibit the oxidase activity of the COX enzyme [55,57], while AOX is resistant [58]. Therefore, even though the affinity of AOX to oxygen is lower than that of COX, AOX can still function as an alternative to maintain respiration under hypoxia. Transgenic manipulation of *AOX1a* revealed an indirect relationship between AOX protein expression and NO in leaves of tobacco under normoxia [59]. AOX reduces electron flow through Complexes III and IV, decreasing the leakage of electrons to nitrite and, thus, suppressing NO accumulation. Excess NO can rapidly react with proteins and other free radicals, forming *S*-nitrosylated and tyrosine-nitrated proteins in addition to producing reactive nitrogen species (RNS) [60,61]. The relationship between AOX and NO scavenging was recently investigated using plants that were subjected to hypoxia or treated with flagellin (flg22) [60,62], as this elicitor also induces NO production [63]. Both flg22 and hypoxia induced *AOX1a* mRNA and protein accumulation in seedlings by at least 6- and 15-fold, respectively [62]. Nitric oxide was more highly produced in *aox1a* antisense line roots under control conditions and in plants exposed to fgl22, while the opposite was found for *AOX1a* overexpressors [62]. In accordance, a more pronounced formation of ROS and RNS in addition to tyrosine-nitrated proteins was observed in the *AOX1a* antisense lines. The opposite effect on NO formation was observed when the plants were subjected to hypoxia, suggesting a discrete role for AOX1a during oxygen deprivation. Under hypoxia, both *AOX1a* overexpressors and WT plants increase NO emission, while this was found to be decreased in *aox1a* antisense lines [60,62]. Treatment with SHAM, an AOX1a inhibitor, circumvents increased NO emission under hypoxia, which points to a direct role for AOX1a in NO production [60,62]. Interestingly, the increased NO emission in *AOX1a* overexpressors during hypoxia did not result in RNS formation or tyrosine-nitrated proteins [60,62]. In addition, O_2_^−^ and H_2_O_2_ content are indirectly related to AOX1a protein content both during hypoxia and reoxygenation [60]. These data suggest that AOX-mediated NO production under hypoxia is protective rather than harmful to the cell [60,62]. Nitric oxide generation mediated by AOX can feed the phytoglobin–NO cycle, which operates under hypoxia to prevent overreduction of the cell and contributes to ATP production [61,64]. In agreement with this hypothesis, an increase in HEMOGLOBIN 1 (HB1), nitrate reductase activity, and ATP content was found in *AOX1* overexpressors under hypoxia [60,62]. The obtained results are more evident in *aox1a* antisense plants than in overexpressor plants, in line with findings showing that the alternative pathway is not induced just by the increase in AOX1a protein levels. The flow of electrons through the alternative pathway may require other upstream ETC components, such as alternative NDs [65]. Taken together, these data reinforce the role of the alternative respiration in keeping RNS and ROS under control during hypoxia stress and reoxygenation, as well as contributing to cellular energy status.

#### 2.2.2. ALTERNATIVE NAD(P)H DEHYDROGENASES

In *Arabidopsis*, ALTERNATIVE NAD(P)H DEHYDROGENASES are encoded by the following small gene families: NDA1–NDA2, NDB1–NDB4, and NDC1 [66]. Type B NAD(P)H DEHYDROGENASES are localized to the external surface of the mitochondrial inner membrane, whereas NDAs and NDC1 reside on the mitochondrial matrix-facing surface of the inner membrane [67]. Here, NDC1 is not discussed due to the lack of information on its role in mitochondrial signaling and stress response. In addition, NDC1 is present at a very low copy number (five copies) per mitochondrion [11] and is also found in the chloroplast [68,69].

##### Internal NADP(H) DEHYDROGENASES

Because the *Km* of NDA1 to NADH is higher than that of Complex I NADH DEHYDROGENASE [70], NDA1 is assumed to be functional only when the concentrations of NADH in the matrix are high [67], such as when plants are grown under high light. The protein content of NDA1 decreases in dark-treated potato leaves, and its transcript abundance responds to the diurnal cycle [71], whereas NDA2 is not responsive to light [72,73]. In this scenario, NDA2 is thought to be mainly functional in heterotrophic tissues [66]. An in silico analysis of the publicly available transcriptomes shows mild expression of *NDA2* in heterotrophic developmental stages, such as during germination [47] (Figure 2) or pollen tube growth [74]. The expression of *NDA1* and *NDA2* is repressed during germination (Figure 2). Recent evidence suggests that neither *NDA1* nor *NDA2* is induced in high-light-treated plants [75]. As responsiveness to light was observed at the protein level, it is possible that *NDA1* is post-transcriptionally regulated in high-light-treated plants. In agreement with this possibility, the phenotype of *Arabidopsis nda1 nda2* double knockdown lines was only apparent in light-growing plants, although no distinction between high-light induction of AOX- and complex IV-mediated dark respiration was observed [67]. When these plant lines were subjected to high-light treatment, photorespiratory, TCA cycle, and hypoxia intermediates accumulated. Due to the apparent lack of transcriptional correlation between *NDA1* and *NDA2*, these results might explain additional independent effects caused by the silencing of both genes. Perhaps individual assessment of *NDA1* and *NDA2* silencing lines would help provide further information, especially regarding *NDA2*. Interestingly, transcriptomic data for the *nda1 nda2* RNAi-silenced line did not indicate major changes in central metabolism or provoke alterations in nuclear gene expression, suggesting that NDAs are important to study the regulation of mitochondrial function but do not elicit major mitochondrial changes by themselves.

Together with AOXs, NDs were hypothesized to be important in plant adaptation to hypoxia [76], although experimental evidence for this is currently lacking. The transcript levels of *NDA1* and *NDA2* are not responsive to hypoxia, but *NDA2* is upregulated in dark-submerged plants (Figure 2). Compared to untreated controls, both were induced in light-submerged plants [53] (Figure 2). Additionally, *NDA1* is downregulated twofold in transgenic plants overexpressing *RAP2.12*, [77], a key transcription factor involved in low-oxygen signaling [78]. These results may suggest a role for internal NDs during submergence, but it does not seem linked to oxygen availability. As other gases have impaired diffusion underwater, INDs may be functional in response to ethylene, CO_2_, or NO.

##### External NAD(P)H DEHYDROGENASEs

Three of the four external NDs (NDB1–4) have been characterized. The protein NDB1 catalyzes Ca^2+^- and pH-dependent NADPH dehydrogenase activity [79,80], while NDB2 and NDB4 oxidize NADH in a Ca^2+^-stimulated and Ca^2+^-independent manner [79]. The expression levels of *NDB3* and *NDB4* are much lower than *NDB1–2* levels throughout development [36]. The expression of *NDB3* and *NDB4* across *Arabidopsis* accessions is not detectable in several ecotypes [81], suggesting that both proteins are unnecessary for photosynthetic tissues or can be functionally substituted by other isoforms. According to an expression search across publicly available datasets, we found that *NDB3* is upregulated at least 30-fold during germination (Figure 2) and 10-fold during anther development [82], suggesting that this protein has a role during heterotrophic stages of development or in non-photosynthesizing tissues. However, it should be considered that *NDB4* is consistently upregulated (at least sixfold) when plants are subjected to high light [75]. Silencing *NDB4* did not decrease external NADH oxidation [83], which is consistent with the findings that most external NADH is oxidized by NDB2 [65]. *Arabidopsis* lines with reduced NDB4 content showed higher NDB2 and AOX1a protein levels and increased total and AOX mitochondrial respiration [83]. Again, this is consistent with the increased respiration when both *NDB2* and *AOX1a* are concomitantly overexpressed [65]. These results show that alterations in *NDB4* transcript levels could lead to altered expression of *NDB2* and *AOX1a*, probably as a compensatory mechanism. Unfortunately, lines with altered NDB4 content have not been further studied, and the possible signaling mechanisms involved are currently unknown.

Both *NDB1* and *NDB2* are the most expressed isoforms at the RNA level [36,81], and, in the proteomic data of isolated mitochondria, a higher average number of copies are observed for NDB1 and NDB2 (590 and 1524 copies per mitochondrion, respectively) [11]. Suppression of *NDB1* caused a mild growth phenotype in *Arabidopsis* plants grown in soil [84] but not hydroponically [85]. Furthermore, these knockdown lines displayed decreased Ca^+2^-dependent NADPH oxidation [84], even though the NADPH or NADP^+^ levels differed between the two studies compared to the WT [84,85]. The fact that no differences were observed in the NADPH/NADP^+^ ratio between the WT and the *NDB1*-suppressed line suggests that NDB1 may not be necessary to remove excess reductants caused by high light or ammonium nutrition [85]. In agreement, NDB1 is not induced by high light (Figure 2), thus indicating that plants can cope with high light or ammonium-induced reductant accumulation by other means. Interestingly, the transcriptional profile of *NDB1* suppressor lines overlaps with that of the ABA INSENSITIVE 4 (ABI4) mutant [84]. The ABI4 protein is a known regulator of organellar retrograde signaling and *AOX* expression [86], whose expression was not altered in *NDB1* suppressor lines. Additionally, in general, the expression of several redox-sensitive genes was regulated differently by ammonium in WT and *ndb1* knockdown plants [85], reinforcing these bypass proteins as essential components of mitochondrial signaling.

The absence of studies that address the combined mutation or overexpression of *NDs* and *AOXs* is surprising. We hypothesize that these combinations may result in extreme phenotypes that make it challenging to analyze the resulting line. Another bottleneck could involve technical issues, including the loss of silencing or loss of overexpression when stacking gene silencing/overexpression cassettes. To our knowledge, a single study combined overexpression of both *NDs* and *AOXs* [65]. The results demonstrated that NDB2 is the major external NADH dehydrogenase in plants since external NADH oxidation was almost absent in *ndb2* knockdown lines. Interestingly, *NDB2* overexpression could not engage the remaining components of the electron transport chain since NADH oxidation was not altered. When the overexpression of *AOX1a* and *NDB2* was combined, NADH oxidation rates increased by up to fourfold compared to single *NDB2* or *AOX1a* overexpressors. These data suggest that increasing the capacity of AOX allows NDB2 protein overexpression to be active. Although plant growth was not affected in these dual overexpressor lines under normal controlled conditions, they lowered the plant tolerance to a combination of drought and moderately high-light stress. These conditions are likely to cause photoinhibition because of both reduced Complex V activity (drought) [87] and overreduction of ETC components (high light) [88], which in turn requires the dissipation of excess reducing power for plant survival. Overall, the described results reinforce the importance of genotype combinations for studying alterations in OXPHOS components. Efforts should be made to produce lines combining silencing or overexpression of NDs, AOXs, and UCPs, as they must work cooperatively in vivo.

## 3. Mitochondrial Retrograde Signaling and Crosstalk with Chloroplasts

Mitochondrial retrograde signaling mechanisms are much better characterized in mammals and fungi than in plants. This reflects that mitochondria are intrinsically connected with several human diseases and metabolic disorders. A major challenge for this topic in plant sciences is the presence of chloroplasts, which necessitate much more attention, as they are a unique feature in green organisms. In addition, chloroplasts are intrinsically connected to mitochondria in the cellular energetic balance, likely leading to distinct and more complex pathways than those of heterotrophic organisms. Thus, it is possible that a “master regulator” of mitochondrial biogenesis does not exist in plants. The history of how retrograde signaling has been studied in plants was recently reviewed [6]. In the subsections below, we discuss the major advances that have recently occurred in plant mitochondrial retrograde signaling and crosstalk with chloroplasts.

### 3.1. Arabidopsis NO APICAL MERISTEM (ANAC) Transcription Factors

A comprehensive search for *cis*-regulatory elements in a subset of genes that are responsive either to impaired mitochondrial function in response to treatments with respiratory inhibitors or to genetic mutation of mitochondrial proteins led to the discovery of a mitochondrial dysfunction motif (MDM) [89]. A set of 24 genes that contain the MDM motif showed strong transcriptional induction in several mitochondrial perturbation experiments, suggesting that these genes represent robust marker genes for mitochondrial dysfunctions [89,90,91]. This set of genes is often referred to as part of the mitochondrial dysfunction stimulon (MDS). Using yeast two-hybrid and electrophoretic mobility shift assays, it was demonstrated that five ANAC transcription factors (ANAC013, 16, 17, 53, and 78) were able to bind to the described MDM motif. The binding of ANAC013 was verified in planta by chromatin immunoprecipitation, and its capacity to upregulate mitochondrial genes was confirmed in ANAC013 gain-of-function plants [91]. A distinct, elegant analysis using forward genetics was conducted, identifying ANAC017 as a regulator of *AOX1a* [91]. Interestingly, both ANA013 and ANAC017 localize to the endoplasmic reticulum (ER) membrane, and the precise mechanism via which an ER protein can induce transcription in the nucleus was carefully investigated. A combination of treatments in lines containing the promoter regions of *AOX1a* fused with a LUCIFERASE reporter with a rhomboid protease and the development of transgenic lines expressing ANAC017 tagged with GFP and RFP (GREEN and RED FLUORESCENT PROTEIN, respectively) in the N- and C-termini, respectively, revealed that the N-terminal portion of ANAC017 moves to the nucleus to regulate gene expression [91]. The regulation of genes that are part of the MDS by ANAC017 was clearly confirmed in *ANAC017* overexpressor and knockdown lines [53,92]. The *ANAC016* gene is located next to *ANAC017* within the *Arabidopsis* genome and is the closest paralog to *ANAC017*, with 71% identity [92]. Retrograde signaling responses are not different in *anac016* knockout mutants compared to WT plants, while the *anac017* knockout line displays almost complete abolishment in the expression of the downstream MDS transcripts [92]. Interestingly, *ANAC016* and *ANAC013* were induced in the *ANAC017* overexpression lines examined in these studies, suggesting that ANAC017 positively regulates *ANAC016* and *ANAC013*. [92]. Electromobility shift assays further experimentally confirmed that ANAC017 binds to the first intron of *ANAC016*. [92]. The identification of ANAC017 as an important regulator of the stress response and mitochondrial gene expression was probably not noticed by researchers because of its steady expression across *Arabidopsis* development and perturbation datasets, as remarked by the authors [91]. In accordance, few datasets show variable amounts of *ANAC017*, and its expression is relatively stable across *Arabidopsis* accessions [81]. However, all the mentioned ANACs (13, 16, and 17) are surprisingly repressed during germination, as opposed to a substantial number of mitochondrial transcripts [47]. Expressing the referred ANACs under various stress-inducing chemicals confirmed the lack of response of *ANAC017*, while *ANAC016* is responsive to methyl viologen (MV), AA, and UV light. A broad response was observed for *ANAC013* except for MV treatment. Analysis of these *ANAC* genes at the transcription level must be carefully interpreted, as they must be post-transcriptionally modified to induce changes upon gene expression.

**Figure 3:**
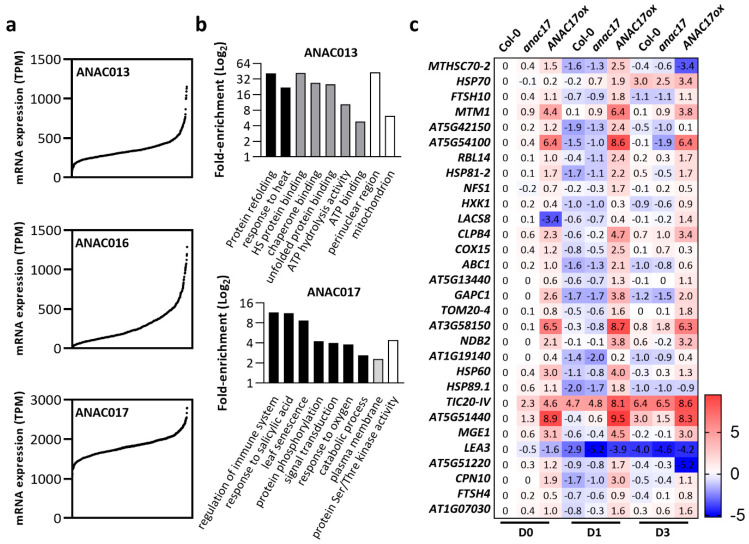
Transcripts co-expressed with *ANAC013* are differentially expressed in *ANAC017* overexpressor lines under normal growth conditions and during senescence. (**a**) *ANAC013*, *16*, and *17* mRNA expression in ascending order across a global collection of 727 *Arabidopsis thaliana* accessions [81]. The dataset was obtained from GEO within the NCBI database. Original transcriptome data were deposited to the GEO at the NCBI under accession number GSE80744. Specific data points are not comparable between graphs. (**b**) Co-expression analysis was conducted individually for ANAC013, 16, and 17 across the accessions within the abovementioned dataset (GSE80744) to obtain the Pearson correlation coefficient. Genes were considered as co-expressed using a Pearson correlation cutoff value ≥ 0.5. A Gene Ontology enrichment analysis was conducted using the co-expressed genes as queries against the complete *Arabidopsis* transcriptome as background. Gene ontologies were considered enriched using a twofold and FDR < 0.05 cutoff. Black bars: biological process, gray bars: molecular function, white bars: cellular compartment. (**c**) A total of 30 mitochondrial targeted genes, as annotated at TAIR Subcellular Prediction, were found as co-expressed with ANAC13. The expression of these 30 mitochondrial genes was searched in Col-0, *anac17*, and ANAC17ox lines during dark-induced senescence [92]. Original datasets used to build this heatmap are available at ArrayExpress under the following accession number: MTAB-8478. Relative expression is expressed as log_2_ fold-change compared to untreated Col-0 plants. For definitions of gene symbols and for a complete description of the gene names shown in the heatmap, please see Table S2. D0: Control plants, D1: senescence in individually darkened leaves for 1 day, D3: senescence in individually darkened leaves for 3 days. Relative expression is expressed as log_2_ fold-change compared to untreated Col-0 plants (D0).

We reasoned that investigating the abovementioned *ANACs* expression in publicly available natural variation datasets [81] would help analyze possible synergistic effects among them (Figure 3). We found that, consistent with previous studies [93], *ANAC017* exhibited a much higher expression than *ANAC016* and *ANAC013* (Figure 3a). The presence of accessions in which *ANAC017* transcripts were almost absent may serve as future tools to examine its function. In these datasets, we also examined whether the genes with the highest Pearson correlation with *ANAC013*, *ANAC016*, and *ANAC017* were involved in mitochondrial metabolism using Gene Ontology (GO) term enrichment analysis. Mitochondrial-targeted transcripts were largely enriched in *ANAC013* co-expressed transcripts in addition to the GO categories “response to heat” and “protein refolding” (Figure 3b). Mitochondrial genes were not overrepresented in *ANAC016* and *ANAC017* co-expressed transcripts. A variety of genes that belong to particular stress responses were enriched in the set of transcripts that are co-expressed with *ANAC017*, including “regulation of immune system” and “leaf senescence” (Figure 3b). These findings are consistent with the reported involvement of ANAC016 and ANAC017 during natural and dark-induced senescence [92,94,95]. Interestingly, most of the mitochondrial transcripts that were found to be co-expressed with *ANAC013* in *Arabidopsis* accessions were downregulated during senescence in WT plants but presented sustained upregulation in *ANAC017* overexpressors (Figure 3c).

Many ANAC TFs regulate leaf senescence [96]; in a recent study, ANAC017 was identified as a negative regulator of natural leaf senescence progression according to the phenotype of an *anac017* mutant line [97]. Interestingly, an early senescence phenotype and a faster decline in chlorophyll levels with leaf age were also observed in *ANAC017* overexpressors [94]. Senescence- and cell death-related transcripts were found to be enriched in *ANAC017* overexpressors, including positive regulators of leaf senescence, such as *ANAC016*, *ANAC029*, *ANAC046*, *ANAC059*, *ANAC087*, and *ANAC092* [94]. The referenced study observed no early senescence phenotype in the *anac017* mutant lines. The conflicting data between the two studies were attributed to the technical approaches used to evaluate senescence and were further addressed in subsequent studies [92]. The authors have followed a distinct methodology to study leaf senescence by darkening only individual attached leaves while keeping the rest of the plant in optimal conditions for plant growth [92]. This approach allowed the dark-induced senescence to be studied in a synchronized way while maintaining systemic communication, mimicking partial shadowing by a neighboring plant [98]. A faster yellowing of individually darkened leaves and consequently reduced chlorophyll content was observed in all the *ANAC017* overexpressors. In addition, there was no consistent difference in the visible senescence rate of chlorophyll loss between the WT and *anac016* or *anac017* knockout lines used in the study. In line with previous findings [94], the authors also found that differentially expressed genes with sustained upregulation in *ANAC017*-overexpressing lines compared to the WT were enriched in GO terms for autophagy-related components and cell death [92]. These studies suggest a broad role for ANAC017 and, consequently, mitochondria in mediating stress and development responses, such as senescence; however, little has been discussed about metabolic regulation under these mitochondrial dysfunctions. Recently, it was reported that the promoter of a TCA cycle enzyme *ACONITASE 3* (ACO3) contains an MDM motif. The expression and protein content of ACO3 are regulated under chemical perturbations of mitochondrial metabolism in an ANAC017-dependent manner [99], which sets the groundwork for future research on the relationship between ANAC017 and metabolic reprogramming within the cell.

### 3.2. PRT6 N-Degron

The N-degron pathways control the half-life of substrate proteins, which is determined by the identity and form of the amino-terminal (Nt-) residue. Different types of N-degron pathways are ubiquitous in living organisms, suggesting that a mechanism for degradation based on identifying the Nt-residue evolved early for regulating protein fate [100]. N-degron pathways work together with the ubiquitin-proteasome system (UPS) through pathway-specific E3 LIGASE N-RECOGNINS that shuttle substrates for ubiquitin-mediated degradation [100]. An Nt-Cys as part of an N-degron can be destabilizing in plants, and, as in mammals, it depends on the presence of both O_2_ and NO in vivo [101]. Researchers identified the PROTEOLYSIS 6 (PRT6) N-degron pathway and GROUP VII ETHYLENE RESPONSIVE TRANSCRIPTION FACTORS (ERFVII) as key regulators of plant oxygen sensing [78,102]. The five *Arabidopsis* ERFVIIs RELATED TO AP (RAP)2.12, 2.2, and 2.3 and HYPOXIA RESPONSIVE ERF (HRE)1 and HRE2 were shown to be substrates for the PRT6 N-degron pathway. ERFVIIs have been shown to regulate responses to many abiotic and biotic factors [103,104,105,106] and aspects of plant development [106,107,108]. In addition, they contribute to regulating a set of genes in response to chemically induced or submergence-stimulated mitochondrial malfunctioning [77], including mitochondrial *DYCARBOXILATE CARRIERS* (*DICs*) [109]

Overexpression of *RAP2.12* (RAP2.12ox lines) resulted in the induction of a set of genes related to oxidative stress in a plant developmental stage-dependent manner [77]. Seedlings and rosette leaves of the *erfVII* line treated with AA showed a partial suppression or complete abolishment in the response of some targets, including *HB1* and *GLUTATHIONE S-TRANSFERASE TAU 24* (*GSTU24*). In addition to the abovementioned transcripts, *RAP2.12* overexpression resulted in a threefold increase in *OUTER MITOCHONDRIAL MEMBRANE PROTEIN 66* (*OM66*), a known marker of mitochondrial retrograde signaling [110]. Notably, *OM66* does contain the MDM motif in its promoter region, and its transcript abundance is affected in both *ANAC017* overexpressor and knockout lines [92,94]. Interestingly, we also found other nuclear-encoded mitochondrial transcripts in the RAP2.12ox datasets by cross-referencing with TAIR Subcellular Prediction. At least 23 and two transcripts were up- and downregulated upon *RAP2.12* overexpression, respectively, including *AOX1b* (5.1-fold induced). In addition, seeds from the *prt6* mutant presented alterations in the mitochondrial transcripts [78] involved in central carbon and lipid metabolisms, such as *QUA-QUINE STARCH* (*QQS*) [111], *GLYCEROL-3-PHOSPHATE ACYLTRANSFERASE 1* (*GPAT1*) [112], and *DUF581 CYCLIN-DEPENDENT KINASE* (*AT1G74940*), among which the latter was shown to interact with SNRK1 [113]. Moreover, *AOX1a* and *NDB3* were downregulated in seeds from the *prt6* mutant [78].

**Figure 4:**
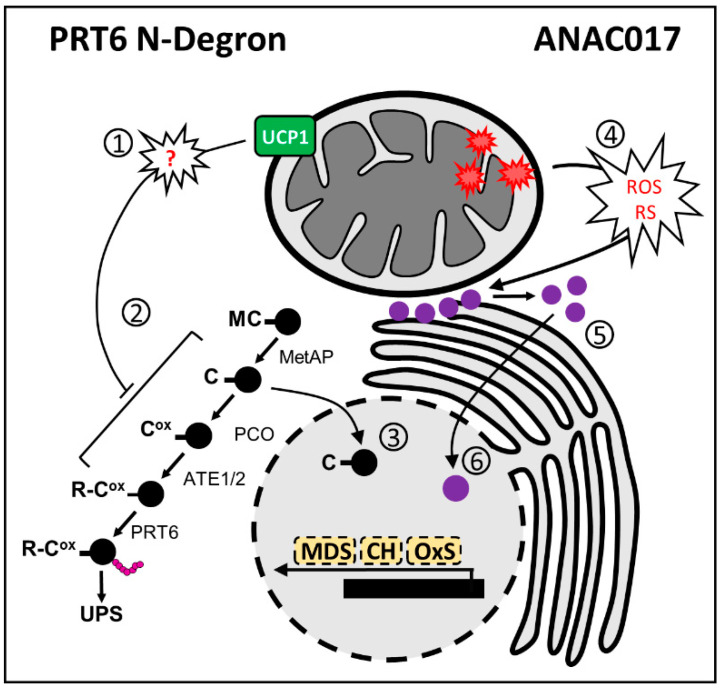
Mechanisms of retrograde signaling in response to alterations of mitochondrial function. **Left panel (PRT6 N-Degron signaling)**: (1) Disturbances of mitochondrial homeostasis by altered UNCOUPLING PROTEIN 1 (UCP1) expression induce the production of yet unknown signals that alter the stability of PRT6 N-Degron substrates. (2) Schematic representation of PROTEOLYSIS 6 (PRT6) N-Degron (Gibbs et al., 2011 [78]). (3) Increased UCP1 activity alters nuclear gene expression via stabilization of the GROUP VII ETHYLENE RESPONSIVE TRANSCRIPTION FACTORS (ERFVIIs). Stable ERFVIIs in the nucleus can induce core-hypoxia (CH) [78] and oxidative stress-related genes (OXs) [77]. A few components of the mitochondrial dysfunction stimulon (MDS) were found to be differentially expressed in lines with altered PRT6 N-Degron depending on the growth stage [77,78]. Black circles indicate protein substrates (ERFVIIs), amino-terminal amino acids are single letter codes, and ox indicates oxidized cysteine. MetAP, METHIONINE AMINO-PEPTIDASE; ATE, ARGINYL TRANSFERASE. **Right panel (ANAC017 signaling)**: (4) Inhibition of mitochondrial respiration at Complex III (using antimycin A) leads to (5) protease cleavage of ANAC017, releasing an amino-terminal (Nt-) fragment that (6) relocates from the endoplasmic reticulum (ER) membrane to the nucleus to activate the expression of MDS components. Reactive oxygen species (ROS) and reductive stress (RS) have been proposed as signals that can mediate ANAC017 release from the ER. In addition to MDS, ANAC017 regulates CH and OxS genes [92,94]. Purple circles indicate the ANAC017 protein, either full-length at the ER or cleaved N-terminal fragment, as soluble and nuclear.

The evidence that ERFVIIs, via the PRT6 N-Degron, could be part of mitochondrial retrograde signaling was further confirmed in collaborative research, including Maia’s and Arruda’s research groups in Brazil with Michael Holdsworth from the University of Nottingham. We showed that UCP1 protein content and activity could alter the stability of ERFVIIs under normoxic conditions [29] (Figure 4, left panel). It was demonstrated that at least part of the UCP1-dependent tolerance to abiotic stress depends on ERFVIIs. Interestingly, the absence of UCP1 in *ucp1* knockdown plants abrogated the accumulation of artificial PRT6 N-Degron substrates in germinating seedlings under regular growth conditions. These results suggest that the presence of UCP1 within the mitochondrial inner membrane is mandatory for ERFVII stabilization, at least during this developmental stage. Due to the dual function attributed to UCP1 [12,14], it is not easy to know precisely why this phenotype was observed. During germination, most ATP is generated by mitochondrial OXPHOS; hence, it is unexpected that mitochondrial respiration is adenylate-restricted, a condition in which UCP1-mediated uncoupling activity would be necessary. It is also unclear how aspartate/glutamate exchange between mitochondria and cytosol could influence ERFVII stabilization. Mammalian adaptation to low oxygen involves the HYPOXIA-INDUCIBLE FACTOR (HIF) oxygen-sensing system [114,115], which is mechanistically different from the plant oxygen-sensing pathway [116]. Interestingly, mammalian HIF1α acts as a repressor of aspartate biosynthesis by suppressing several key aspartate-producing proteins, including the mitochondrial GLUTAMIC-OXALOACETIC TRANSAMINASE-1 (GOT1) [117]. The simple addition of aspartate to the culture medium is sufficient to relieve the HIF1α-dependent repression of tumor cell proliferation. Thus, data from mammals suggest that aspartate transport across the mitochondrial membrane, as part of the malate–aspartate shuttle, is pivotal for adaptation to mitochondrial dysfunction, which might involve UCP1 in plants. Our understanding of the involvement of the PRT6 N-Degron in mitochondrial retrograde signaling is in its first stages. We hope that, in the next few years, more data will be generated, allowing us to obtain more comprehensive insight into the roles of ERFVIIs in coordinating mitochondrial function.

### 3.3. Crosstalk between ANAC and PRT6 N-Degron in Mitochondrial Retrograde Signaling

It is currently unknown whether the PRT6 N-Degron (Figure 4, left panel) or UCP1 is mechanistically connected to ANAC017-based signaling (Figure 4, right panel). However, some observations make the crosstalk between these pathways or an independent complementary action highly attractive. There is extensive overlap in the transcriptomic footprint of ANAC017-mediated signaling induced by Complex III inhibition with antimycin with that of hypoxia [118]. In addition, an artificial PRT6 N-Degron substrate is stabilized in plants treated with the AOX inhibitor SHAM [29]. In line with this hypothesis, the *HRE2* gene, which is among the five ERFVIIs and part of the mitochondrial dysfunction stimulon, is induced by at least 50-fold by *ANAC017* overexpression [92,94]. Additionally, *HRE1* is consistently downregulated in *ANAC017* overexpressor lines [94]. The transcriptome of UCP1ox tobacco lines [20,119] shows extensive changes that are not controlled by the PRT6 N-Degron pathway, suggesting either pleiotropic rearrangements or crosstalk with other signaling mechanisms. The response observed in UCP1ox tobacco lines includes alterations in protein biosynthesis and light reactions of photosynthesis, which are primarily affected by mitochondrial dysfunctions [120].

**Figure 5:**
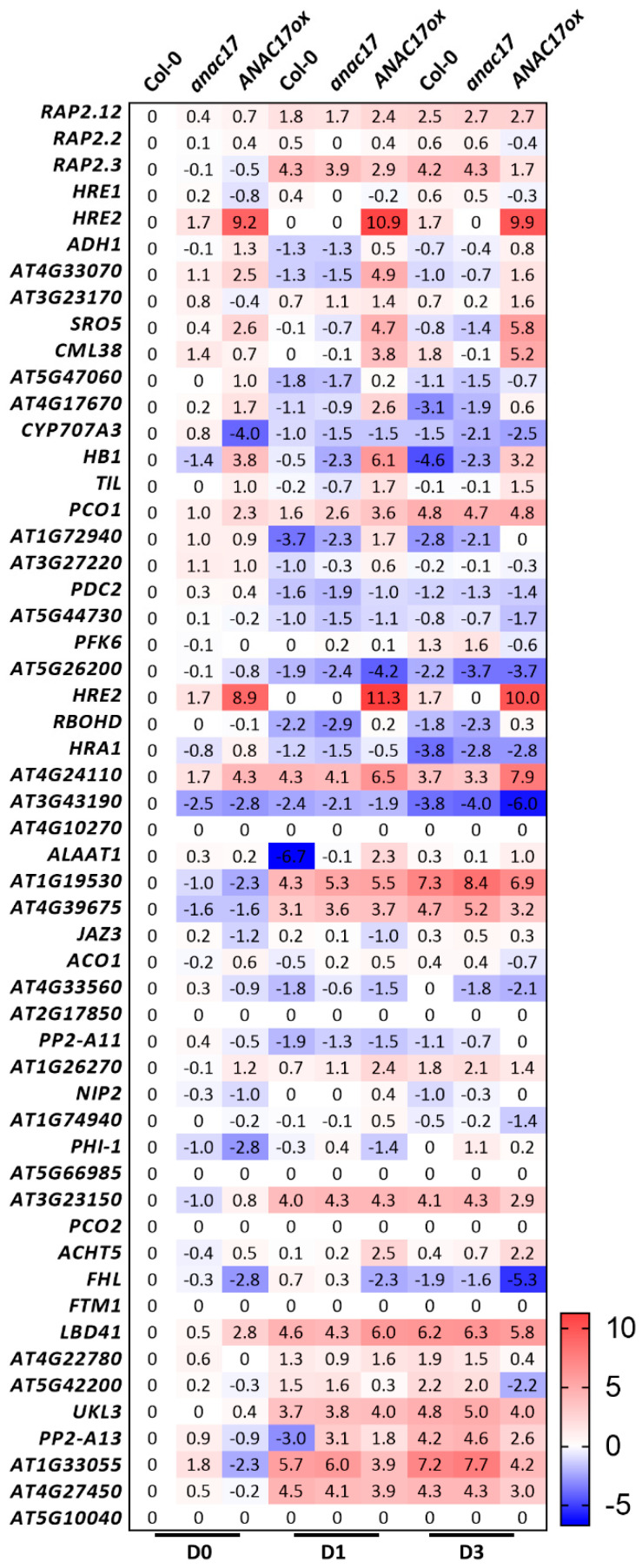
Expression of core-hypoxia genes in ANAC017 lines during senescence in individually darkened leaves. The expression of the previously defined core-hypoxia genes [121] was investigated in publicly available datasets of Col-0, *anac017*, and *ANAC017* overexpressor lines during senescence in individually darkened leaves [92]. Original datasets used to build this heatmap are available at ArrayExpress under the following accession number: MTAB-8478. D0: control plants, D1: senescence in individually darkened leaves for 1 day, D3: senescence in individually darkened leaves for 3 days. Relative expression is expressed as log_2_ fold-change compared to untreated Col-0 plants (D0). For definitions of gene symbols and for a complete description of the gene names that are found in the heatmap, please see Table S3.

To further investigate this overlap, we examined the behavior of *ERFVIIs* and core-hypoxia transcripts [121], which are primarily affected in the *prt6* mutant [78] and *ANAC017* overexpressor and knockdown lines during senescence [92] (Figure 5). Among the *ERFVIIs*, *RAP2.12*, *RAP2.3*, and *HRE2* were induced following dark incubation. Although the expression of *RAP2.12* gradually increased in individually dark-incubated leaves from WT and *anac017* lines, the observed induction occurred more rapidly in ANAC017ox on the first day of dark incubation. The *RAP2.3* gene was otherwise rapidly induced by at least 15-fold during the experiment, which was attenuated in the ANAC017ox lines. In the opposite direction, *RAP2.2* and *HRE1* did not appear to be part of the senescence response. Both *RAP2.2* and *HRE1* were significantly attenuated for at least one timepoint in the experiment with the ANAC017ox lines. The response of the *ERFVIIs* following dark incubation showed no difference between the WT and *anac017* lines except for *HRE2*. In addition to the *ERFVIIs*, at least 21 core-hypoxia transcripts were differentially expressed between WT and ANAC017ox under normal growth conditions considering a twofold cutoff (Figure 5). Additionally, 27 and 30 core-hypoxia transcripts were differentially expressed after 1 and 3 days of dark incubation, respectively, in WT plants. Interesting patterns were found for some transcripts, including *ALCOHOL DEHYDROGENASE 1* (*ADH1*) and *HB1.* The *ADH1* gene was downregulated after 1 day of dark incubation in both WT and *anac017* lines, and its expression was partially recovered after 3 days, with levels comparable to those in nontreated plants. In contrast, ANAC017ox lines presented higher *ADH1* expression at day 0, and the corresponding transcript was induced after dark incubation. In addition, *HB1* is down and upregulated in *anac017* and ANAC017ox, respectively, in nontreated control plants. Downregulation was exacerbated after 1 day of dark incubation in these lines compared to the WT. Moreover, after 3 days of dark incubation, downregulation was observed in the WT and *anac017* lines compared to control plants, while a least eightfold induction was detected in the ANAC017ox line. We tested a similar approach by searching for MDS transcripts in the *prt6* and RAP2.12ox lines [77,78]. The MDS genes were searched for in microarray data of the *prt6* mutant, which limited our analysis since not all the transcripts were available for searching. This analysis is also difficult because ERFVII activity preferentially occurs at specific stages of development [77]. We found four MDS transcripts that were differentially expressed between *prt6* and WT seeds. The genes *CONSTANS-like 9* (*COL9*) and *HRE2* were upregulated in *prt6*, while *AOX1a* and *CYTOCHROME P450, FAMILY 81, SUBFAMILY D,* and *POLYPEPTIDE 8* (*CYP81D8*) were downregulated. As expected, the differences in seedlings differed from those in seeds, as only *HRE2* and a *MATE EFFLUX FAMILY PROTEIN* (*AT2G04050*) were upregulated in *prt6*. Moreover, the RAP2.12ox line presented an increase in *OM66* and *HRE2* transcript abundance, while no MDS genes were downregulated in this line. In summary, at least at the transcript level, no strong crosstalk was found when examining the MDS genes in PRT6 N-Degron lines, while the analysis performed in the other direction, in which the core-hypoxia transcripts were searched in ANAC017 lines, revealed a stronger correlation.

These data reinforce the existence of possible crosstalk between PRT6 N-Degron and ANAC017 signaling, although there is no experimental confirmation thus far. In addition to expression data, ANAC017-based signaling has been shown to independently contribute to submergence and hypoxia tolerance [53,122]. It was recently shown that ANAC017 directly recruits a signaling cascade involving the plant hormones ethylene and auxin [123]. In parallel, it was previously shown that ethylene enhances *HB1* expression, which in turn reduces NO levels and stabilizes ERFVIIs [124]. Ethylene also increases ROS production and triggers a mitochondrial retrograde signaling cascade involving the upregulation of *ANAC013* and *AOX1a* during seed germination to break dormancy [125]. Thus, ethylene may be the prime signal or at least a good candidate that contributes to the integration of mitochondrial dysfunction and hypoxia signaling.

## 4. Mitochondria–Chloroplast Crosstalk

Given the interaction in metabolism between chloroplasts and mitochondria, it is not surprising that signaling pathways from these organelles may share several components. All the mechanisms and proteins discussed in this review are linked to chloroplast metabolism. Mitochondrial respiration bypasses, including internal and external NDs and AOX1a, are discussed here regarding their importance in consuming reductants generated by chloroplasts under high light. In addition, *UCP1* overexpressors and mutants have altered photosynthetic performance [21,23,24]. Mutant *ucp1* lines have impaired photorespiration performance due to the lack of NAD^+^ regeneration within the mitochondrial electron transport chain, while *UCP1* overexpressors presented increased carbon assimilation, stomatal conductance, and starch content under both regular and stressful conditions. In addition, both ANAC017 and PRT6 N-Degron markedly affect photosynthesis. The first was shown to repress the expression of chloroplast-targeted transcripts. In this case, many of the associated genes encoding photosynthesis components, including several *LIGHT HARVESTING CHLOROPHYLL A/B BINDING PROTEINs* (*LHCBs*) and a starch degradation-related *BETA AMYLASE 6 FERRIC REDUCTION OXIDASE 7*, important for chloroplast iron acquisition and photosynthetic efficiency [126], were downregulated in the ANAC017ox lines [94]. Interestingly, the direct relationship between mitochondrial dysfunction and repression of chloroplast genes was previously observed in a meta-data analysis of public transcriptomes [120]. Furthermore, ANAC017 directly binds to RADICAL INDUCED CELL DEATH 1 (RCD1) [127], a nuclear protein that participates in diverse stress and developmental processes that alter both mitochondrial and chloroplast functions [128,129,130]. These results demonstrated that RCD1 integrates the response to mitochondrial- or chloroplast-derived ROS to induce the expression of MDS genes, including *AOX*.

Concerning its involvement in chloroplast metabolism, the PRT6 N-Degron follows the same pattern observed for other mitochondrial regulators. Etiolated *Arabidopsis* seedlings of WT and PRT N-Degron mutants were analyzed for subsequent transfer to light under hypoxic or normoxic conditions [131]. After transfer to light under normoxia, the total chlorophyll levels were much lower in *prt6* mutants compared to WT. In addition, WT seedlings presented a smaller chlorophyll content under hypoxia following transfer to light, which was not observed in *erfVII* lines. In addition, the expression of several chloroplast forms of heme-synthases was greatly repressed in WT by hypoxia both in the dark and following transfer to light, while it was also constitutively repressed in the *prt6* line independent of oxygen availability. Furthermore, this repression was not observed in *erfviiI* or *prt6 erfvii* mutants, indicating that downregulation is achieved by stabilized ERFVIIs [131]. This mechanism that links oxygen sensing to chlorophyll biosynthesis was shown to be widespread in natural populations of flowering plants [132]. A direct relationship was observed between protochlorophyllide levels and the altitude at which plants are grown. Following these findings, *Arabidopsis* accessions from contrasting altitudes display altitude-dependent ERFVII activity and accumulation [132].

In addition to the transcriptional regulation of gene expression, chloroplasts and mitochondria are intimately linked via posttranscriptional control of protein activity and by the exchange of intermediate metabolites produced within the mitochondria, especially by the tricarboxylic acid cycle. How photosynthesis is affected by mitochondrial metabolism was particularly focused upon by the Brazilian researchers Adriano Nunes-Nesi and Wagner Araújo from the Federal University of Viçosa [133,134,135]. These researchers showed that manipulation of TCA cycle enzymes using reverse genetic approaches dramatically affects carbon assimilation. Inhibition of *ACONITASE 1* (*ACO1*) or *MALATE DEHYDROGENASE* (*MDH*) resulted in 50% and 20% increases in net carbon assimilation, respectively. Thiol-disulfide redox exchange, which can be controlled by THIOREDOXINS (TRX), is a widely distributed post-translational modification crucial to plant metabolic regulation. This issue, not specifically mitochondrial TRXs, was extensively investigated by the Brazilian researcher Danilo Daloso from the Federal University of Ceará [136]. Thioredoxins contribute to correct protein folding and to the (de)activation of target proteins. The first clue linking TRXs to the regulation of TCA cycle enzymes emerged from proteomics studies in which members of the TCA cycle of plant mitochondria were found to bind to TRX [137,138].

There is evidence showing that TRX regulates TCA cycle enzymes, including both SUCCINATE DEHYDROGENASE (SDH) and FUMARASE (FUM) in vivo [139], and others, such as CITRATE SYNTHASE (CS) and ISOCITRATE DEHYDROGENASE (IDH), as well as AOX [136]. Among the chloroplast targets of the TRX system are proteins involved in starch biosynthesis, ATP synthesis, and the chlorophyll biosynthetic pathway, in addition to enzymes of the Calvin–Benson (CB) cycle [140]. The complexity of interorganellar redox communication is evidenced by the number of targets that have already been identified to be TRX-regulated in both mitochondria and chloroplasts [136]. Recently, a putative mitochondrial-localized TRX (TRX h2) was shown to be involved in germination and stomatal conductance without altering the CO_2_ assimilation rate under ambient O_2_ conditions [141]. However, the *trx-h2* mutant presented a decreased photosynthetic rate under high photorespiratory conditions. This protein was later identified in the microsomal fraction but not in the mitochondrial fraction [142]. This result agrees with various studies in which it became apparent that organellar functions influence a wide range of processes outside of the organelles themselves [143]. Examples include the influence of plastid TRXs on respiratory and mitochondrial metabolism on plastid redox balance [136]. In accordance, *trx-h2* mutants showed substantial changes in the level of metabolites related to photorespiration and decreased transcript levels of the photorespiratory complexes *GLYCINE DECARBOXYLASE* (*GDC*) and *SERINE HYDROXYMETHYLTRANSFERASE* (*SHMT*). In silico analyses suggest that GDC subunits have a high probability of forming various disulfide bonds, thus bearing the potential for redox regulation. These data suggest that TRX h2 plays an important role in the redox regulation of mitochondrial photorespiration. However, there is likely much to learn about TCA cycle regulation by post-translational modifications since several TCA cycle enzymes are candidates for additional regulation, such as phosphorylation, acetylation, or both [144,145]. Detailed in vivo studies using knockdown mutants and metabolic profiling are necessary to establish which of these post-translational modifications is physiologically important. In addition, given the broad range of targets that TRXs can reduce and the processes in which they are involved, an interesting approach in the near future would be to focus on determining the specific targets of the mitochondrial TRX enzymes in vivo.

## 5. Inner Membrane Transporters

In addition to the molecular signaling and respiratory activity described in the past sections, mitochondria can adapt their function and composition by regulating the exchange of substrates with the cytosol [146,147]. Although the mitochondrial outer membrane is permeable to small molecules (with a molecular mass of less than 4–5 kDa) [148,149,150], the mitochondrial inner membrane is impermeable to polar molecules. In the latter case, only nonpolar molecules, such as O_2_ and CO_2_, can diffuse passively through the lipid bilayer. The passage of specific hydrophilic compounds across the MIM is mediated by a large and diverse inventory of transporters, most of which belong to the MITOCHONDRIAL CARRIER FAMILY (MCF) of proteins [151]. The molecules transported by MIM carriers are highly variable in size and structure, ranging from the smallest possible substrate H^+^ (via lipid shuttling) to large compounds such as NAD^+^ and COENZYME A. For a comprehensive review of the diversity of MIM carriers, we recommend the recent review written by Adriano Nunes-Nesi and collaborators [147]. The employment of reverse genetics to provide insights into the in vivo function of MIM carriers is discussed in addition to an extensive in silico analysis regarding the circumstances in which these carriers are differentially expressed [147]. Biochemical characterization and functional studies of the MIM carriers were also reviewed recently [152]. Here, we briefly discuss a potential role and future perspective in studying mitochondrial carriers considering cellular signaling. 

Recent strong evidence shows that plant DICARBOXYLATE CARRIER 2 (DIC2) is a malate–citrate antiporter in both isolated mitochondria and proteoliposomes. This protein plays a critical role in the coordination of anaplerotic metabolism with consequences for assimilatory and catabolic pathways between the mitochondria and other cellular compartments [153]. It was shown that DIC2-mediated malate/citrate transport affects leaf respiration in the dark, especially in the light-to-dark transition. It was proposed that alterations in the performance of the *dic2* mutant could be linked to an altered distribution of TCA cycle metabolites between mitochondria and cytosol, which leads to changes in metabolic and NAD redox states in these compartments. To observe any rapid and transient changes that occur in the NAD redox state during a sudden transition from light to dark, the fluorescent protein biosensor Peredox-mCherry [154], which reports cytosolic NADH/NAD^+^, was used. No differences in NADH/NAD^+^ ratios between WT and *dic2* knockdown plants were found during illumination; however, upon transfer to darkness, the expected decline in NADH/NAD^+^ ratios was significantly slower in *dic2* compared to the WT [153]. Fluorescent genetically encoded biosensors have been shown to be a valuable tool, especially for studying membrane transporters. Utilizing fluorescence overcomes some of the limitations of destructive analytical methods to capture the status of different redox couples in the cell, such as (i) the need for cell tissue homogenization, meaning that many samples are needed if dynamic changes are to be followed, (ii) the risk of artifactual changes in the redox status of these molecules during their extraction, and (iii) the differences between individual cells or cell compartments, which cannot be resolved even though those differences are often of particular interest [155]. This system allows for minimally invasive live monitoring of local redox status in living cells, tissues, and organisms. The sensor protein is genetically encoded and can be precisely targeted to specific subcellular compartments using the cellular targeting machinery and appropriate signal sequences for organelle import. Using this technique, combined with ratiometric fluorescence microscopy or fluorimetry, the redox potential can be quantified in living plant cells, and several applications of this technique have been developed [155,156,157,158].

Recently, the functional characterization of two *Arabidopsis* lines that exhibited reduced expression of two genes encoding mitochondrial NAD^+^ carriers (*NAD^+^ TRANSPORTERS 1* and *2*; *NDT1* and *NDT2*) indicated that among the cellular processes affected by impaired NAD^+^ transport are stomatal function, conductance, and density [156]. Accordingly, combined genetic and pharmacological approaches to manipulate cellular and subcellular NAD^+^ dynamics demonstrate that NAD^+^ negatively correlates with the stomatal number in *Arabidopsis* cotyledons. The referred NAD^+^ mutants, along with NAD^+^-treated WT seedlings, displayed reduced stomatal number, and, although NAD^+^ administration impacted the stomatal number in cotyledons of WT seedlings, NAD^+^ carrier mutants were insensitive to this treatment. Additionally, it was demonstrated that mutations in NAD^+^ transporters interfere with biometric parameters of stomata and pavement cells, including stomatal area and width. The results provide clear and elegant evidence that NAD^+^ dynamics are important for modulating the expression of stomatal biogenesis-related genes. Considering that the regulation of several stomatal biogenesis pathway genes depends on ABA metabolism and that the *ndt1* and *ndt2* mutants show delayed seed germination [156,159], a classic ABA phenotype, it was hypothesized that the NAD^+^ carrier mutant phenotype is linked to deregulated ABA metabolism and/or signaling [157]. Accordingly, ABA-related genes were upregulated in NAD^+^ carrier mutants, while increased levels of ABA were observed in an NAD^+^-dependent manner in seedlings carrying the sensor ABAleon2.1. These results indicate the direct involvement of mitochondrial NAD^+^ transport in modulating ABA levels and signaling, thus impacting stomatal function. In addition to fluorescent-encoded biosensors, metabolic analyses, especially isotope labeling to deduce metabolic fluxes, have been recently used to identify specific metabolic fluxes in roots subjected to hypoxia [160]. Identifying metabolic fluxes instead of individual metabolite pools is of special interest when studying mitochondrial inner membrane carriers, especially because they do not consume or produce new substrates but rather mediate their translocation and access to different metabolic enzymes. In addition, a nonaqueous fractionation technique has been successfully employed to accurately determine in planta metabolite content in plastids and the cytosol. Nevertheless, methods to perform mitochondrial measurements are under development and must be improved [151]. In conclusion, employing these recent techniques may soon result in new discoveries on the impact of mitochondrial transporters on cellular metabolism, as well as the precise transport property capacity of MIMs.

## 6. Manipulating Mitochondrial Metabolism to Improve Plant Resistance against Environmental Stresses

Since mitochondrial metabolism exerts a vital role in plant survival and performance by itself and by communicating/influencing other organelles, manipulating the associated pathways would provide a groundbreaking tool for crop improvement. The mitochondrial set of chemical reactions modulates several processes and outcomes, such as abiotic and biotic stress tolerance, as well as plant development and performance. Most players (proteins) and responses usually overlap since the signals are intertwined. For instance, it was shown that chemically restricting the COX and AOX pathways in rice simultaneously leads to lower photosynthesis rates, higher ROS, and decreased drought and salt stress tolerance [161]. Therefore, the COX and AOX pathways proved essential for combating stress and maintaining optimal photosynthetic activity.

*ALTERNATIVE OXIDASE* is among the most intensively investigated mitochondrial genes that exhibit different physiological roles, assuring its merit [162]. The expression of *AOX1* has been linked to alleviating excess reducing power from impediments to normal mitochondrial electron transport chain activity [163]. Therefore, AOX1 has been shown to mitigate several unfavorable environmental conditions [164,165,166,167,168] and enhance photosynthetic performance [48,169,170,171,172]. In this context, the relationship between proline catabolism, an ROS-producing pathway activated during stress, and AOX (isoforms 1a and 1d) activity was explored in plants under stress [46]. The results indicate that *AOX1a* and *AOX1d* are upregulated in response to proline, helping the ETC cope with the extra redox imbalance burden. They both function to limit oxidative stress, thereby enhancing photosynthetic activity and facilitating plant recovery from osmotic stress. A synergistic negative effect is also observed when both genes are disrupted.

Salt stress also compromises the efficiency of photosynthesis, limits carbon assimilation, and reduces plant growth and crop productivity [173]. This stress increases ROS production and induces osmotic and ionic stresses, which hamper water absorption and photosynthesis efficiency. The application of external chemical compounds that target mitochondria has been identified as an alternative for increasing crop tolerance to stresses [174]. Applying chemical priming for this purpose, a chemical library of the RIKEN Natural Products Depository (NPDepo) was screened, and a new promising compound (FSL0260) was identified and tested in *Arabidopsis* [175]. The FSL0260 molecule binds and inhibits Complex I of ETC, activating an alternative respiration pathway by upregulating *AtAOX1a* and *AtNDB4*. As a major outcome, FSL0260 reduces ROS accumulation and enhances plant tolerance to salinity. Comparative proteomic profiling revealed distinct responses of wheat root tips and mature tissues to saline stress [176]. Translation of proteins related to the TCA cycle and OXPHOS, including cytosolic MDH and ATP SYNTHASE subunits, decreased in abundance in root tips, indicating a significant effect of salt stress on energy production. Higher energy requirements may be due to the highly energy-consuming processes necessary to guarantee ion homeostasis, osmotic adjustment, and ROS defense [177]. The TCA cycle seems to be drained during stress, and ATP synthase subunits were decreased in abundance in root tips—the most stress-affected tissue. In *A. thaliana,* mutants defective in mitochondrial TRANSCRIPTION TERMINATION FACTOR 27 (mTERF27) were more sensitive to saline stress, and loss of gene function impaired mitochondrial gene expression and overall development under salt stress [178].

Another important component related to mitochondrial functioning is γ-aminobutyric acid (GABA). This metabolite regulates the cytosolic pH, limits ROS production, adjusts N metabolism, and can support mitochondrial respiration when the TCA cycle is hampered, which overall represent important roles in the adaptation of plants to stress [179]. Under saline stress, leaves from wheat (*Triticum aestivum*) plants exhibited reduced activity of mitochondrial pyruvate transporters and pyruvate dehydrogenase (PYRUVATE DEHYDROGENASE COMPLEX, mtPDC, and 2-OXOGLUTARATE DEHYDROGENASE COMPLEX, mtOGDC) subunits, indicating impaired oxidation of mitochondrial pyruvate. Therefore, key TCA enzymes are physiochemically inhibited by salt, hindering mitochondrial respiration [180]. The authors observed that the GABA shunt was activated by salt treatment, providing an alternative carbon source for mitochondria and promoting an increase in leaf respiration [180]. Therefore, this provides another step to understanding avenues to upgrade crops [181].

Photosynthesis is also inextricably related to mitochondrial metabolism since mitochondria and chloroplasts act coordinately to optimize energy metabolism in light [134,182]. Drought strongly perturbs photosynthesis due to stomata closure, which occurs to reduce water loss but also limits the availability of CO_2_ for the Calvin–Benson cycle [183,184]. This may generate an energy imbalance, in which ATP and NADPH are generated at a slower pace by the chloroplast ETC than their consumption by the CB cycle. As most of the ATP demands in mature leaves are used for sucrose synthesis and phloem loading, both limited during drought stress, intracellular ATP content increases, and mitochondrial respiration becomes adenylate-restricted [87]. During severe drought, additional biochemical limitations may further reduce photosynthesis beyond that resulting from CO_2_ limitation [185]. In this regard, the evaluation of *N. tabacum* aox knockdown plants showed that this protein maintains respiration and preserves photosynthetic capacity during moderate drought [41]. The *aox* knockdown plants displayed a 10–15% lower photosynthetic rate at high irradiance due to stomatal limitation resulting from disrupted NO homeostasis within the guard cells [186]. Thus, AOX is a necessary ETC component to maintain mitochondrial respiration during photosynthesis. In the absence of this electron sink, respiration is slowed, accompanied by changes in the composition of the photosynthetic apparatus that consequently compromise photosynthetic capacity. These results also indicate that AOX is a mandatory electron sink that supports photosynthesis when the CB cycle is compromised, which occurs in conditions such as drought [41]. In contrast, when *AOX* was overexpressed in tobacco plants, an enhanced tolerance to drought was observed, which was related to overcoming biochemical limitations [187].

Later, AOX was shown to be critical to maintain respiration and prevent widespread oxidative damage and loss of function of both mitochondria and chloroplasts [40]. Accelerating the rates of mitochondrial respiration in the light (R_L_) under extreme drought depends upon AOX activity, partly due to a concomitant loss of COX capacity. The rate of mitochondrial R_L_ was shown to determine the reduction state of the photosynthetic ETC in the chloroplast. This supports the function of AOX in maintaining chloroplast homeostasis. When AOX is lacking, the energy imbalance accelerates the accumulation of carbonylated mitochondrial and chloroplast proteins, which positively correlates with an accelerated loss of function of mitochondrial and chloroplast ETC protein activities. The mentioned study provides a definitive example in which AOX is critical in maintaining respiration and preventing widespread oxidative damage and loss of function of mitochondria and chloroplasts [40].

As previously mentioned, TRXs are ubiquitous proteins that also regulate AOX and enzymes of the TCA cycle, among others. In the absence of a functional mitochondrial TRX system, other mechanisms occur to maintain redox homeostasis under stresses, such as drought and salt. This includes increased activities of enzymes that are part of redox metabolism and increased concentrations of secondary metabolites. Additionally, TRX is important for stomatal function, allowing higher stomatal closure during salt stress and better recovery of stomatal conductance following rehydration; this is a key factor for maintaining plant growth [188]. Lastly, perturbations in mitochondrial homeostasis influence a variety of cellular processes that rely on mechanisms and components that are not fully identified; TRX, with its diverse targets, is one of them [188].

Another important line of cell defense involves antioxidant enzymes. For instance, SUPEROXIDE DISMUTASE (SOD) participates in a highly efficient ROS-scavenging system, catalyzing the dismutation of superoxide anions into H_2_O_2_ and O_2_ in different cell compartments [189,190]. A mitochondrial iron SOD (*TdFeSOD4A-1*) was upregulated in durum wheat (*Triticum durum*) subjected to salt, drought, cold, and ABA treatments. The manganese-type SOD *TdMnSOD2B*, also localized in the mitochondrion, was strongly expressed in wheat roots and leaves under cold stress [191]. This study provides insights into promising candidate genes to enhance wheat resilience, including those associated with mitochondria.

As already stated, mitochondria also influence biotic stresses. Complexes I and III are the primary redox centers in the ETC that can leak single electrons to oxygen and form ROS [6]. Mitochondria are also linked with the oxidative burst during plant–pathogen interactions. Thus, a pathogen can suppress plant immunity by inhibiting mitochondrial function, such as with *Pseudomonas syringae,* by expressing HOPG1, an effector protein [192]. In addition, the investigation of the regulatory network of rice–*Xanthomonas oryzae* pv. *oryzae* interaction led to the discovery that this pathogen hijacks the host immune system by restricting mitochondria-generated ROS [193]. *Xanthomonas oryzae* suppresses plant miR159a.1, preventing its targets, such as *RICE IMMUNITY REPRESSOR 1* (*RIR1*), from being silenced. Subsequently, elevated RIR1 associates with the NADH-UBIQUINONE OXIDOREDUCTASE subunit of mitochondrial Complex I to limit ROS production, thereby subverting rice immunity to favor the invading pathogen. It is worth noting that mitochondrial proteins are key players in ROS balance, and that they are crucial for plant resistance to diverse factors. Interestingly, mitochondrial stress may also induce plant resistance through chromatin changes, providing epigenetic memory for future generations to better cope with previous adversities [194]. An investigation beginning in the intracellular cascades triggered by the perception of a pathogen until epigenetic memory establishment resulted in the finding that mitochondrial stress plays a role in plant-induced resistance. The authors observed broad-spectrum mitochondrial stress-induced resistance to pathogens caused by epigenetic changes. Uncovering this process mechanistically opens up possibilities to strengthen the plant system, which is extremely relevant for agriculture [194].

A healthy mitochondrial network is also essential to adjust this far-from-balanced oxidative environment in the cell. Thus, when stress cannot be alleviated, defective mitochondria are removed by autophagy, which is a process called mitophagy. Plants employ mitophagy to recycle damaged mitochondria during stress and development (such as de-etiolation) [195]. Loss of mitochondrial ΔΨm is among the red flags that trigger the control system. Studying *Arabidopsis*, Ma et al. (2021) [195] showed that uncoupler treatments (2,4-dinitrophenol (DNP) and carbonyl cyanide *p*-(tri-fluromethoxy) phenyl-hydrazone (FCCP)) cause loss of mitochondrial membrane potential, followed by activated “eat-me” signals given by the compromised organelle. Then, autophagosomes selectively engulf the damaged mitochondrion. Additionally, a clustered mitochondrial family protein called FRIENDLY (FMT) was essential for uncoupler-induced mitophagy. Upon damage, FMT is recruited, thus regulating mitochondrial dynamics. Lastly, mitochondrial quality control and turnover are crucial for good cell performance and, consequently, for the organism’s adaptation to environmental changes. Moreover, mitochondrial recycling and chloroplast biogenesis during de-etiolation show how the two organelles are intertwined to support developmental transition in plants [195].

Another example regarding plant development is a mitochondria-localized ATP-dependent metalloprotease that belongs to the FILAMENTATION TEMPERATURE-SENSITIVE H (FTSH) family, NEEDLE1 (NDL1), which maintains a reproductive meristem redox status and auxin homeostasis. The protein NDL1 regulates maize inflorescence architecture and is necessary for maize growth and yield under field conditions and high temperatures [196]. Furthermore, mitochondria were found to play a role in root elongation via energy production and interaction with hormonal signaling and ROS homeostasis. *Arabidopsis* mutants with a knockdown in the *SUCCINATE DEHYDROGENASE ASSEMBLY FACTOR 2* gene presented ROS-mediated auxin hypersensitivity, causing pH-dependent root elongation [197]. These metabolic changes can inhibit root elongation at low pH [197]. Another relevant example is the maize MITOCHONDRIAL RIBOSOMAL PROTEIN DEK44 (DEK44), which is necessary for kernel development [198]. Defective DEK44 mutants resulted in small kernels and delayed development. The lack of DEK44 affects the expression of genes from the mitochondrial respiration chain subunit, reduces the function of ETC complexes, and inhibits cell proliferation. Thus, mitochondrial oxidative phosphorylation is arrested during embryo and endosperm development [198]. In addition, disruption of the *EMPTY PERICARP 24* and *25* (*EMP24* and *EMP25*) genes inhibits intron splicing and, consequently, the synthesis of proteins involved in Complex I assembly and activity, thus impairing energy supply and seed development [199].

Interestingly, mitochondrial activities may also be related to the integrity of cell walls, the first layer of cell defense against biotic and abiotic stressors [200]. For example, an identified CELLULOSE SYNTHASE COMPLEX (CSC) inhibitor named C17 can reduce cellulose production by perturbing CSC activity. Growth defects caused by C17 can be suppressed by modulation of mitochondrial activity and mitochondrial retrograde signaling, ultimately controlling cell-wall integrity maintenance [200]. Additionally, mitochondrial genes have also been investigated to improve fruit quality. In this regard, the apple PYROPHOSPHATE-ENERGIZED PROTON PUMP (MDMA12) was shown to have important functions related to apple fruit acidity, an important factor influencing its nutritional quality and taste [201]. By performing ectopic expression and overexpression of MDMA12 in *Solanum lycopersicum* “Micro-Tom” (MT) tomato and apple calli, respectively, the authors found that MDMA12 increased malic acid content, inducing malate synthesis in the mitochondria and the expression of malate transporters.

In photosynthetic cells, mitochondria share the title of a powerhouse with plastids. A considerable amount of mitochondrial respiration is not intended to generate energy by ATP production. These alternative pathways to transport electrons uncoupled from ATP synthesis require the activity of NDA-, NDB-, and NDC-type noncoupled NADH and NADPH dehydrogenases, AOX, and UCP, as described in the previous sections. Several functions of uncoupled mitochondrial respiration have been described in plants [61], including thermogenesis, fruit ripening, plant protection from abiotic and biotic stresses, and metabolite production or catabolism [15,61]. In addition, alternative and uncoupled respiration contributes to better performance and growth of agricultural crops [163,202,203]. All these functions are likely to result from their more general function, such as providing steady fluxes to support cellular biosynthesis. Several findings indicate that alternative and uncoupled respiration involve ROS and NO scavenging, regulation, and homeostasis. Additionally, they induce signaling events and have a role in stress defense and biosynthesis and catabolism processes, enabling plants to adapt to changing environments [15,61], as seen from the examples discussed.

Photosynthetic efficiency was also positively linked to a mitochondrial SMALL HEAT-SHOCK PROTEIN 23.6 (HSP23.6) in MT tomato plants subjected to various stresses. Micro-tom plants overexpressing *sHSP23.6* maintained photosystem activity, the electron transport chain, and CO_2_ assimilation when subjected to high-temperature cycles [204] and flooding [205]. In addition, these plants had a better modulation of the antioxidant system and higher lycopene content in fruits when subjected to hypoxia and moderate water deficit [206,207]. More recently, the same research group compared the effects of drought imposed during flowering on the photosynthetic activities of MT wildtype plants and those exhibiting silenced or overexpressed *sHSP23.6* [208]. The MT-sHSP23.6-overexpressing plants maintained their photosystem electron flow, energy generation, and chlorophyll index. They also tolerated water deficit conditions longer, showing lower physiological effects, especially concerning photochemical reactions, thus contributing to better physiological and biochemical performances [209]. Water deficit can increase leaf temperature, inducing oxidative and thermal disturbances [210,211]. The sHSP acts mainly as a molecular chaperone, folding, localizing, repairing, and degrading proteins in living organisms, which helps to maintain cellular homeostasis under various stresses. This constitutes another interesting example of how manipulating mitochondrial genes can generate plants with better photosynthetic efficiency to cope with unfavorable environmental conditions [212].

Photorespiration (C2 cycle) is another counterintuitive process. Although considered an energetically wasteful process, photorespiration is an integral part of plant primary metabolism, and impairing its flux leads to negative impacts on cellular processes. The prime function of the oxidative photosynthetic carbon cycle is to rescue the glycolate-2-P that is produced by the oxygenase activity of RUBISCO. This cycle involves large machinery and relies on chloroplasts, peroxisomes, and mitochondria. Photorespiration ultimately leads to massive production of NADH in the mitochondrial matrix through GLYCINE DECARBOXYLASE (GDC, a multienzyme system) activity [213]. Interestingly, GDC is inhibited by high NADH/NAD^+^ ratios, protecting mitochondria from overreduction if NADH recycling is not optimal [214]. Moreover, a severe reduction in leaf-carbohydrate status was found in a *HYDROXYPYRUVATE REDUCTASE 1* (*hpr1*) knockdown mutant with moderately impaired photorespiration in plants that also showed altered GDC activity [215]. This protein represents another mitochondrial system that is interlinked with other cell organelles and processes allowing an improvement on cell function and protection against stresses. Moreover, overexpression of the GDC H- and L-protein stimulates photosynthesis and plant growth [216,217]. Altogether, these examples show how mitochondria sense the environment and share a broad connection with other cell organelles and processes, impacting various responses that can ultimately improve plant resilience and performance.

## 7. Conclusions and Perspectives

It is safe to conclude that major advances have been made in the last 10 years in understanding the function of plant mitochondria. This was possible due to the implementation of state-of-the-art molecular biology techniques, such as forward genetics, the development of proteomics for the study of isolated mitochondria, the application of respirometric studies on isolated mitochondria, and the development of fluorescent and nonfluorescent biosensors to infer in vivo and ex vivo parameters related to mitochondrial metabolism and protein stability. Because of these major advances, exciting new questions arise while old questions persist. Among the resilient questions is the in vivo function of UCPs in uncoupling mitochondrial respiration. Experiments designed to answer whether UCP1 uncouples respiration by proton transport or exchanges aspartate/glutamate with the cytosol are urgently needed. They will help explain how UCP1 can influence the stability of the PRT6 N-Degron substrates. On the other hand, the well-characterized AOX1a can be used as a tool for understanding the biological function of INDs and ENDs through the generation of double overexpressors or knockdowns. In addition, CRISPR technologies offer advances for studying these bypasses, as this tool can completely knock out the referred genes. The most exciting advances have been made in discovering new mitochondrial retrograde signaling mechanisms, such as those involving ANACs and the PRT6 N-Degron pathway. Extensive studies of ANACs have provided solid evidence during the past 10 years that ANACs contribute to cellular adaptations to mitochondrial dysfunction. According to the function of ANAC in regulating the TCA cycle enzyme ACO3, we should discover how this mechanism contributes to energy-related processes in the future. We hope to witness advances in the contribution of ERFVII stabilization by the PRT6 N-Degron concerning mitochondrial function and energetic metabolism. In addition, a question that arises regarding the regulation of core-hypoxia genes by ANACs and mitochondrial and oxidative stress-related genes by ERFVIIs is how these two mechanisms interact. An emerging field is the study of mitochondrial transporters using stable isotope-labeled metabolomics and genetically encoded fluorescent biosensors. These technologies allowed for the comprehensive study of fundamental processes mediated by NAD^+^ and dicarboxylate transporters and how these transporters influence overall cellular metabolism. As a result of these major advances, mitochondrial-related metabolism and signaling have been used as tools for crop improvement. Exploring the energy bypasses, mitochondrial transporters, and TRXs, in addition to the described ANAC and PRT6 N-Degron signaling mechanism, can clearly contribute to plant resilience to abiotic stresses. Moreover, several mitochondrial components not particularly discussed in this review were also proven to be important tools for developing plants more adapted to environmental stresses.

## Data Availability

Not applicable.

## Supplementary Materials

The following supporting information can be downloaded at https://www.mdpi.com/article/10.3390/ijms231911176/s1.
